# Evaluation of the Stellae-123 prognostic gene expression signature in acute myeloid leukemia

**DOI:** 10.3389/fonc.2022.968340

**Published:** 2022-08-17

**Authors:** Adrián Mosquera Orgueira, Andrés Peleteiro Raíndo, José Ángel Díaz Arias, Beatriz Antelo Rodríguez, Mónica López Riñón, Claudio Cerchione, Adolfo de la Fuente Burguera, Marta Sonia González Pérez, Giovanni Martinelli, Pau Montesinos Fernández, Manuel Mateo Pérez Encinas

**Affiliations:** ^1^ Department of Hematology, University Hospital of Santiago de Compostela, Santiago de Compostela, Spain; ^2^ Department of Hematology, Tomelloso Hospital, Ciudad Real, Spain; ^3^ Unit of Hematology, IRCCS Istituto Romagnolo per lo Studio dei Tumori (IRST) “DinoAmadori”, Meldola, Italy; ^4^ Department of Hematology, MD Anderson Cancer Center, Madrid, Spain; ^5^ Department of Hematology, Hospital La Fe, Valencia, Spain

**Keywords:** leukemia, transcriptome, machine learning, survival, risk, prediction

## Abstract

Risk stratification in acute myeloid leukemia (AML) has been extensively improved thanks to the incorporation of recurrent cytogenomic alterations into risk stratification guidelines. However, mortality rates among fit patients assigned to low or intermediate risk groups are still high. Therefore, significant room exists for the improvement of AML prognostication. In a previous work, we presented the Stellae-123 gene expression signature, which achieved a high accuracy in the prognostication of adult patients with AML. Stellae-123 was particularly accurate to restratify patients bearing high-risk mutations, such as *ASXL1, RUNX1* and *TP53*. The intention of the present work was to evaluate the prognostic performance of Stellae-123 in external cohorts using RNAseq technology. For this, we evaluated the signature in 3 different AML cohorts (2 adult and 1 pediatric). Our results indicate that the prognostic performance of the Stellae-123 signature is reproducible in the 3 cohorts of patients. Additionally, we evidenced that the signature was superior to the European LeukemiaNet 2017 and the pediatric clinical risk scores in the prediction of survival at most of the evaluated time points. Furthermore, integration with age substantially enhanced the accuracy of the model. In conclusion, Stellae-123 is a reproducible machine learning algorithm based on a gene expression signature with promising utility in the field of AML.

## Introduction

Risk stratification in acute myeloid leukemia (AML) has been extensively improved during the last decade. The identification of recurrently mutated genes and cytogenetic anomalies has been of high prognostic and therapeutic significance in patients diagnosed with this disease. Most of the currently used AML risk stratification guidelines, like the European Leukemia Net (ELN) risk classification (1), were elaborated using this information. However, those classifications are based primarily on different retrospective data about cytogenetic analyses and a limited number of mutations, and don’t take into account the genomic complexity of AML and the interaction between different drivers. In recent years, several biomarker panels using next-generation sequencing (NGS) of multiple recurrently mutated or aberrantly expressed genes have been proposed to facilitate improved prognostic stratification.

The development of new risk stratification algorithms using mutational data has been the focus of some recent efforts (**Table 1**). In one of these, *Gerstung et al.* (2017) created an AML knowledge bank which involved data from thousands of patients. The utility of this data bank to predict individual outcomes (such as remission, relapse and mortality probabilities) was proved. Furthermore, their results suggest that personally tailored management decisions could reduce the number of allogeneic hematopoietic stem cell transplants (alloHCT) by 20–25%, while maintaining overall survival rates (2). Another recent effort was presented by *Sherve et al.* (2019), who constructed a novel prognostic model by implementing machine learning (ML) algorithms on clinical, cytogenetics and mutational data from AML patients (3). This algorithm achieved great concordance with reality in 5 different AML cohorts.

**Table 1:** Summary of the molecular predictors of AML survival described in the main text.

Study	Analysis Details and Results
*Gergstun et al. (2017)* (2)	• Analyzed a database of 1,540 AML patients with mutation and cytogenetics annotation • Developed a multistate model for clinical outcome prediction that can be used to support treatment decision (e.g., alloHCT)
*Sherve et al. (2019)* (3)	• Analyzed a database with 3,421 AML patients with cytogenetics and mutation annotation for 44 genes • Developed a ML predictor using the XGBOOST algorithm which achieved high precision in the prediction of survival
*Roushangar et al. (2019)* (4)	• Analyzed gene expression profiles of 2,213 AML patients, finding transcriptomic correlations with surrogate markers of mortality in AML (e.g., age)
*Walker et al. (2021)* (5)	• Analyzed 268 patients with cytogenetically normal AML who were treated with intensive regimes • Identified a 10-gene signature predicting patient relapse
*Ng et al. (2016)* (6)	• Developed a leukemia stem cell 17-gene signature, which was highly prognostic in different AML subtypes (N= 907)
*Mosquera et al. (2021)* (7)	• Analyzed gene expression profiles from two different cohorts (N=562, N=137) • Developed a machine learning survival predictor based on a 123-gene signature

Gene expression data is another valuable source of information in order to risk stratify AML patients (8). Transcriptomic changes are associated with different mutations, cytogenetic aberrations and changes in signaling pathways (**Table 1**). Additionally, gene expression can be linked to other prognostic factors, such as biological age. In this line, *Roushangar et al.* performed a comprehensive gene expression analysis from 37 studies and identified gene expression changes associated with surrogate prognostic markers in AML, such as age and molecular subtypes (4). Furthermore, *Walker et al.* identified a 10-gene signature that was strongly predictive of patient relapse in cytogenetically normal AML (5). This was an important finding because these patients are currently classified as intermediate risk (1), demonstrating that this is a very heterogeneous group where an improved risk stratification for clinical decision-making is of the utmost need (9). In another effort, *Ng et al.* created a 17-gene expression score that was highly prognostic in five independent cohorts, and this contributed to a more accurate prediction of early therapy resistance (6). Therefore, it becomes apparent that transcriptomic information can be used to refine risk stratification in AML.

Recently, we presented Stellae-123, which is a machine learning (ML) model based on a gene expression signature capable of providing personalized survival predictions in adult patients with AML (7). Stellae-123 predictions were precise and added significant prognostic information to those patients with high-risk mutations. Stellae-123 contains 123 variables, including the expression of 121 genes, and achieved c-indexes of 0.723 in the training set and 0.700 in the test set, indicating a high reproducibility of the personalized risk prediction. These results are in line with other risk models based on ML with mutational data described so far in the field (6), which emphasizes the possibility of improving risk stratification in AML by implementing artificial intelligence.

In the present study, we aimed to evaluate the prognostic utility of Stellae-123 in patients from 3 different AML cohorts, including adult and pediatric patients. Our results indicate that this signature is reproducible using RNAseq data and can outperform the precision of current risk stratification guidelines in the field.

## Methods

### Data source and cohort characteristics

For this study, we used data from 3 AML cohorts (BeatAML, AMLCG-2008 and the pediatric TARGET AML). The BeatAML programme was aimed to better understand genetic or transcriptional markers and mechanisms of drug sensitivity and resistance in AML (10). BeatAML was composed mostly of adult patients (>17 years of age). This project was developed in a cohort of 672 primary specimens from 562 patients with AML, and extensive functional and genomic analyses on these samples was performed. The TARGET AML cohort was aimed to fully characterize the genomics of pediatric AML (11). It includes fully characterized cases, including gene expression data. Finally, the AMLCG-2008 cohort was composed of 396 adult AML patient samples from a clinical trial that compared the dose-dense regimen S-HAM (Sequential High Dose Cytosine Arabinoside and Mitoxantrone) versus standard double induction in AML patients (12). Risk stratification data was available for all patients. Adult cohorts classified patients according to the ELN-2017 risk stratification recommendations (1), whereas pediatric patients were classified according to the clinical risk score (11).

### Gene expression profile, mutation data and statistical methods

Gene expression values were normalized to FPKM values and cohort-related batch effects were adjusted using ComBat (13). Then, we mapped microarray probes from Affymetrix arrays and RNAseq transcripts to Ensembl gene identifiers. Afterwards, we selected those Stellae-123 transcripts whose expression was measured in the RNAseq protocols of the 3 cohorts. 69 genes were obtained, which were the basis for downstream signature analysis (**Supplementary Table 1**). Similarly, limited mutation annotation was available from the same cohorts, which was used to evaluate their relationship with gene expression risk groups.

Random forests were built to predict survival in the largest cohort (BeatAML, n = 334), and this model was used to obtain survival predictions from the remaining two cohorts. The discriminative capacity of this model was evaluated using bootstrapped Harrel’s c-indexes with 500 cycles. The precision of the predictors was evaluated using time-dependent areas under the curve (AUCs) derived from cross-validated cox survival models. For these calculations, cross-validation was performed with the bootcv algorithm and 500 cycles. In each cycle, 75% of samples were used for training and 25% for testing. In the particular case of BeatAML, we used random forest out-of-bag predictions as input for the cox survival models, in order to reduce the risk of overfitting during the training phase of the model.

## Results

Baseline characteristics of the three cohorts are represented in **Table 2**.

**Table 2:** Baseline Characteristics of patients included in the three cohorts.

	BeatAML	AMLCG 2008	TARGET AML
**N**	334	199	144
**Age**	61 [2-87]	55 [18-74]	9.42 [0.38-22.55]
**Male/Female**	54.80%/45.20%	–	51.03%/48.97%
**ELN-2017 Favorable**	30.60%	38.18%	–
**ELN-2017 Intermediate**	32.14%	25.63%	–
**ELN-2017 Adverse**	37.80%	36.89%	–
**Clinical Risk ScoreFavorable**	–	–	50.74%
**Clinical Risk ScoreIntermediate**	–	–	43.38%
**Clinical Risk ScoreAdverse**	–	–	5.88%
**Relapsed AML**	3.39%	0%	0%
**Secondary AML**	15.54%	11.70%	–

### Validation of the Stellae-123 gene expression signature

**Figure 1:**
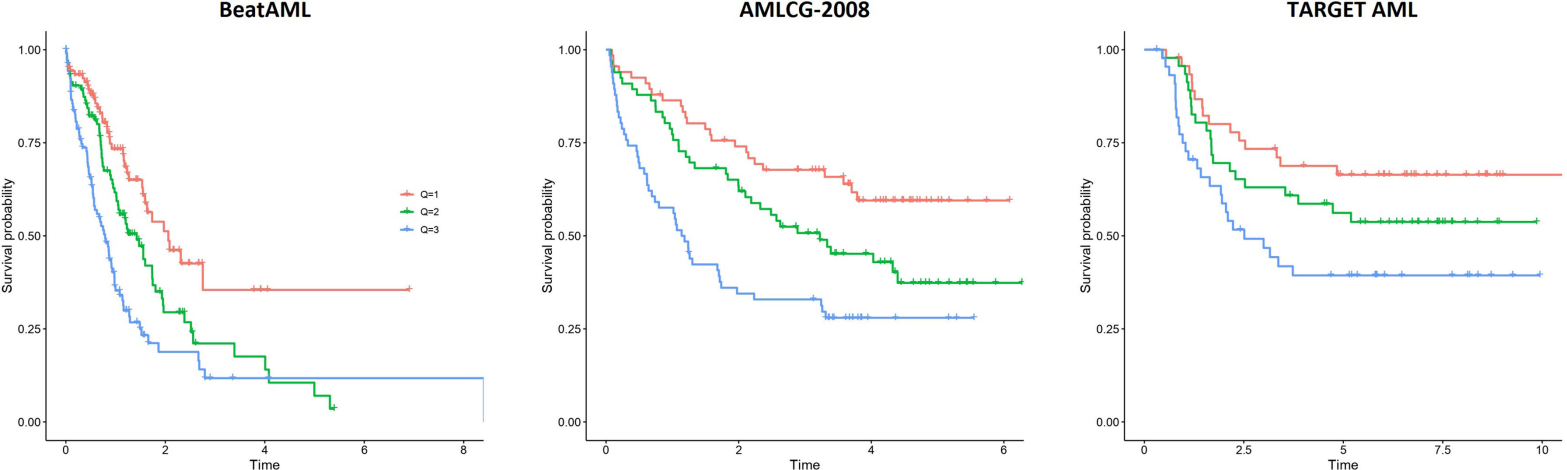
Patient survival according to the tertiles of risk predicted by Stellae-123 in the BeatAML, AMLCG-2008 and TARGET AML cohorts.

A survival predictor based on random forests was built in the BeatAML cohort using Stellae-123 gene expression data. A c-index of 0.635 was obtained after internal cross-validation. We then tested this model in the other two cohorts, AMLCG-2008 and TARGET AML, achieving c-indexes of 0.645 and 0.598 respectively. In **Figure 1**, a representation of patients by tertiles of predicted survival for the different datasets can be consulted. These balanced groups of patients exhibit differences in survival curves corresponding to their expected survival outcomes according to Stellae-123. Afterwards, we evaluated the precision of our model over time, specifically at 6 months, 1 and 2 years after diagnosis (**Table 3**). The AUCs at 1 year were 68.51%, 69.40% and 74.92% for the BeatAML, AMLCG-2008 and TARGET AML cohorts, respectively. Overall, our findings indicate good performance metrics in the three cohorts, and particularly in pediatric patients.

**Table 3:** Comparison in survival prediction between ELN-2017 classification and the pediatric Clinical Risk Score (CRS) with the Stellae-123 model over time.

	Beat AML	AMLCG 2008	TARGET AML
GEP Random Forestc-index	63.55	64.48	59.84
GEP: AUC 6 months	66.45	70.07	75.51
GEP: AUC 12 months	68.51	69.40	74.92
GEP: AUC 24 months	67.57	69.22	60.55
ELN2017: AUC 6 months	59.19	59.09	–
ELN2017: AUC 12 months	57.09	63.64	–
ELN2017: AUC 24 months	65.17	60.96	–
CRS: AUC 6 months	–	–	67.07
CRS: AUC 12 months	–	–	69.25
CRS: AUC24 months	–	–	62.74

### Evaluation of Stellae-123 vs standard risk stratification scores

We compared the precision of this signature with ELN-2017 and the pediatric clinical risk score in the prediction of survival using c-indexes and time-dependent AUCs (6 months, 1 year and 2 after diagnosis). Surprisingly, we observed that the performance of the ELN-2017 classification was low in the BeatAML cohort (Cox bootstrapped c-index, 0.379). Apparently, this was driven by the bad outcomes that intermediate-risk patients had in this cohort (**Supplementary Figure 1A**). A better performance was observed in the AMLCG-2008 dataset, but still inferior to the Stellae-123 classifier (Cox bootstrapped c-index, 0.601). In this case, intermediate-risk patients exhibited better outcomes than expected (**Supplementary Figure 1B**). Time-dependent AUCs also indicate that Stellae-123 is superior to the ELN-2017 classification in the 2 adult cohorts, and that this improvement in survival prediction is sustained over time (**Table 2**).

**Figure 2:**
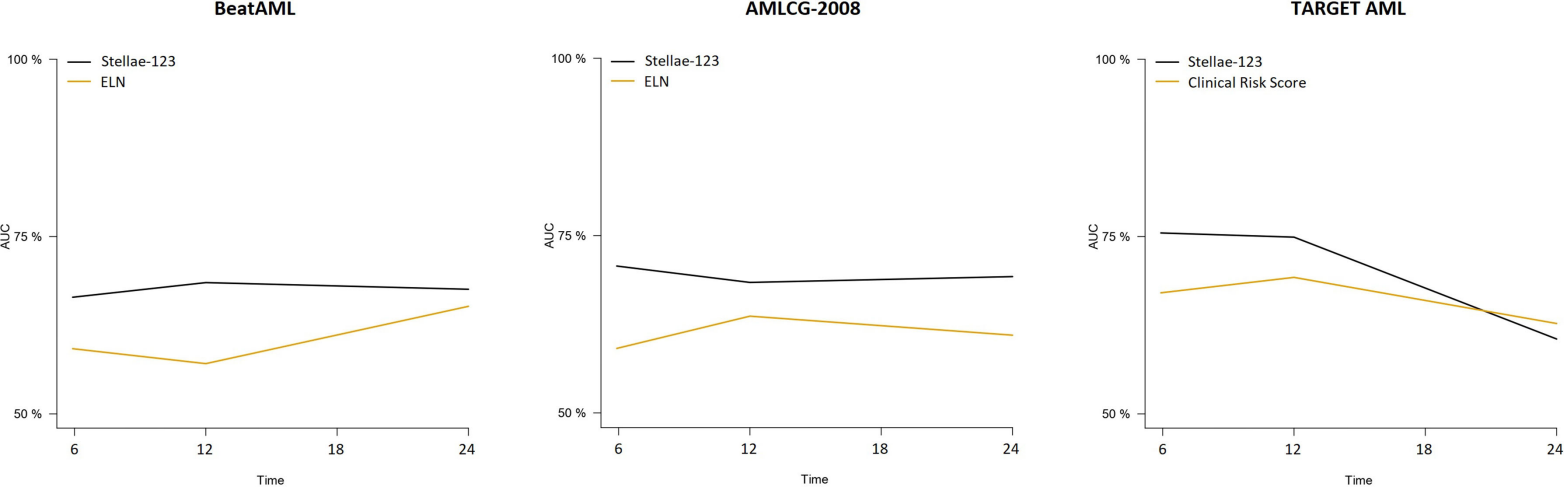
Time-dependent AUCs at 6, 12, 18 and 24 months for the Stellae-123 signature across all the cohorts. For comparison, the performance of the ELN-2017 in the BetaAML and AMLCG-2008 cohorts is shown. In the pediatric TARGET AML cohort, the performance of the pediatric clinical risk score was plotted.

In the TARGET AML pediatric cohort, we observed a better performance of the Clinical Risk Score in terms of c-index (bootstrapped Cox c-index, 0.634), but time-dependent AUCs indicate that our model improved the predictive capacity until the second year after diagnosis. We must mention that the only evaluated time point in which our model was inferior with respect to the current standard models was in the prediction of mortality in the pediatric TARGET AML cohort at 2 years from diagnosis. At this moment, our model obtained an AUC of 60.5%, compared with a 62.7% of the pediatric clinical risk score. (**Figure 2**). We observed that the predictive capacity of Stellae-123 worsens with time, in line with previous findings indicating that the prognostic weight of gene expression variables is greater during the first year after diagnosis (14) (**Figure 2**). The best performance of Stellae-123 at earlier time moments might be driven by the apparently low proportion of patients assigned by the Clinical Risk Score to the high risk group (5.8%), and thus the transcriptomic model might be more suitable to identify early mortality (**Supplementary Figure 1C**), particularly among patients assigned to the standard risk group (**Supplementary Figure 2**).

### Distribution of mutations according to Stellae-123 risk groups

We evaluated the distribution of the most common driver mutations in AML across the different tertiles of risk according to the Stellae-123 risk model. In adult patients, we observed an increase of low risk or neutral risk mutations (*CEBPA*, *NPM1* and *DNMT3A*) in the low and intermediate risk tertiles defined by Stellae-123, whereas high risk mutations (*ASXL1*, *RUNX1*, *TP53* and *U2AF1*) were more common in the high risk tertile (**Supplementary Tables 2** and **3**). Nevertheless, we found that some patients with high risk mutations were assigned to the intermediate or even low risk tertile in both the BeatAML and AMLCG-8 cohorts. This occurred in 26.92% (BeatAML) and 16.67% (AMLCG-8) of patients with *ASXL1* mutation; 36.36% (BeatAML) and 33.33% (AMCLG-8) of patients with *RUNX1* mutation; 10.71% (BeatAML) and 9.09% (AMLCG-8) of patients with *TP53* mutation; and 26.67% (BeatAML) and 14.28% of cases with *U2AF1* mutation. In pediatric patients, we also observed an increased distribution of low and neutral risk mutations (e.g. *CEBPA* and *NPM1*) in the lower and intermediate risk tertiles, but this cohort was devoid of high risk mutations for evaluation (**Supplementary Table 4**).

### Evaluation of prognostic scores considering patient age

Age is a variable deeply associated with survival in AML (15, 16). Therefore, we evaluated the performance of the models including this covariate. We observed clear improvements in AUCs for the BeatAML and TARGET AML cohorts, but not for the AMLCG-2008 cohort (**Table 4**), a finding which is probably related to the fact that these were homogeneously fit adult patients recruited in a clinical trial. Notably, we also observed an improved performance of the gene expression signature plus age model compared with the ELN-2017 and pediatric clinical risk score models, particularly in the prediction of survival during the first year after diagnosis (**Table 4**). Interestingly, by adding age to our model in the TARGET AML pediatric cohort, we achieved an AUC of 87.1% in the prediction of mortality at 6 months from diagnosis, a great improvement over the same model without age (AUC 75.5%) and notably superior to the current clinical risk score plus age at that same time point (AUC 78.1%).

**Table 4:** Comparison in survival prediction between ELN-2017 classification and the pediatric Clinical Risk Score (CRS) with the Stellae-123 model over time including age.

	Beat AML	AMLCG 2008	TARGET AML
GEP + Age: AUC 6 months	75.08	70.68	87.09
GEP + Age: AUC 12 months	74.74	69.75	77.17
GEP + Age: AUC 24 months	73.21	70.55	66.75
ELN2017 + Age: AUC 6 months	72.88	64.47	–
ELN2017 + Age: AUC 12 months	71.77	68.66	–
ELN2017 + Age: AUC 24 months	74.83	67.52	–
CRS + Age: AUC 6 months	–	–	78.14
CRS + Age: AUC 12 months	–	–	72.01
CRS + Age: AUC 24 months	–	–	67.36
ELN2017 + Age: BS 6 months	0, 153	0, 135	–
ELN2017 + Age: BS 12 months	0, 214	0, 179	–
ELN2017 + Age: BS 24 months	0, 186	0, 234	–
GEP + Age: BS 6 months	0, 148	0, 130	0, 014
GEP + Age: BS 12 months	0, 203	0, 178	0, 094
GEP + Age: BS 24 months	0, 192	0, 220	0, 202
CRS + Age: BS 6 months	–	–	0, 014
CRS + Age: BS 12 months	–	–	0, 096
CRS + Age: BS 24 months	–	–	0, 201

AUCs and Brier Scores (BS) are provided.

## Discussion

Despite significant advances in our understanding of the impact of mutations on overall survival, established AML risk stratification guidelines, like ELN-2017 classification, are based primarily on a limited number of genomic drivers. Furthermore, the outcomes of many patients in the ELN-2017 intermediate risk groups are indeed poor and heterogeneous (median survival: 0.7 - 1.6 years) (17), evidencing that there is at least a subgroup of patients for whom a reclassification is critical. Prognostic stratification needs to be improved by taking into account the complexity and the interaction between genomic drivers. Gene expression signatures have been proposed to be effective biomarkers and have promising potential for clinical applications (18). In this context, we have developed a new prognostic score based on gene expression analysis (*Stellae-123*) which achieved high discriminative power in the prediction of survival among adult AML patients (7). In the present work, we have validated the prognostic precision and discriminative power of the *Stellae-123* gene expression signature in 3 external cohorts, and we prove that this signature can predict overall survival with greater precision than the ELN-2017 classification. Furthermore, our preliminary data also supports an improved performance over the pediatric clinical risk score.

During the last years, compelling evidence has been gathered about the possibility of optimizing risk stratification in AML using novel sources of biological information. Indeed, several studies indicated how different gene expression and mutational patterns can improve our comprehension about disease evolution. Some of these studies tried to incorporate expression parameters to the ELN-2017 classification, but failed to achieve a substantial improvement in performance (19). Other works refined the ELN-2017 risk classification by including new mutated genes. One of this achievements was the delineation of a “very favorable” AML subgroup composed of patients with inv(16)/t(16;16) or biallelic *CEBPA* mutations, and a “very adverse” AML subgroup composed of patients with *TP53* mutations and a complex karyotype (20). A refinement of such classification has been presented which applies machine learning algorithms to cytogenetics and mutational data from thousands of AML patients (21). These results enabled the identification of new patients subgroups who do not benefit from alloHCT in first complete remission, and others who should be considered for inclusion in clinical trials due to their infamous prognosis. In the same line, *Sherve et al.* (2019) communicated a novel prognostic model using artificial intelligence that incorporated clinical, cytogenetic and mutational data. This model achieved very precise survival predictions, largely outperforming the ELN-2017 classifications (3). In another very interesting study, *Docking et al.* (2021) elegantly demonstrated how RNAseq can be used as a tool to characterize prognostic gene expression signatures and to identify mutations and structural variants in one single test. Indeed, their data supports the idea that nearly a quarter of patients with AML can be reassigned to a different risk group when considering transcriptomic data (22). These findings, along with ours, strongly support the need for the implementation of standardized, transcriptome-based prognostic signatures for patients diagnosed with AML, a fact which will require the incorporation of new workflows in our molecular biology laboratories.

We observed a relatively high number of patients with high risk mutations that were assigned to an intermediate or even low risk group by Stellae-123. This is in line with previous findings about the prognostic heterogeneity of these mutations in myeloid malignancies, and particularly those affecting *TP53*. *Bernard et al.* (2020) observed that monoallelic *TP53* hits did not influence prognosis in myelodysplastic syndrome patients, whereas multi-hit somatic events in *TP53* were independently associated with adverse outcome (23). This is in line with data from *Montalban-Bravo et al.* (2020) reflecting that the prognostic role of *TP53* can be influenced by variant frequency and genomic context (24). Thus, variations in gene expression might reflect changes caused by complex somatic events, and these transcriptomic shadows in some cases might be a better reflection of functionality than the determination of the mutation itself. As a consequence, gene expression models might be complementary to those based on cytogenetics and mutational parameters.

Future perspectives for Stellae-123 should be to test its prognostic value in the context of prospective clinical trials and to evaluate its usefulness as a guideline for transplant decision. With respect to alloHCT, a prospective clinical trial would need substantial logistical and economic resources. An interesting alternative approach would require the retrospective evaluation of pediatric and adult patients in the real-world who were transplanted according to the current clinical practice recommendations. This could shed light about new groups of patients who might have a substantial benefit from alloHCT or alternative approaches, a fact which has been previously suggested by others (2). Additionally, such a tool could be useful to identify high-risk patients for early inclusion in clinical trials. Finally, an increasing number of risk stratification models based on genomic data are being presented. Future approaches should try to evaluate the different models, and they might be able to derive a meta-model that outperforms the capacity of each individual tool.

The present study has some limitations. Firstly, only 69 out of the 121 transcripts included in the original Stellae-123 signature were available for analysis. Nevertheless, the performance of the 69-gene classifier was above that of the ELN-2017 and the pediatric clinical risk score classifications. An analysis including the entire set of genes would be expected to provide even more accurate predictions. Secondly, additional information about patient baseline characteristics (e.g., performance status metrics, transplant vs non-transplant candidates…) would be useful to refine mortality predictions. Additionally, future studies should also try to inspect the cause of death (i.e., whether the deaths were leukemia-related, transplant-related or not related to the hematological malignancy). Finally, the development of homogeneous databases representing all types of AML patients and treatment protocols, accompanied by an extensive set of molecular data, would be a remarkable milestone in the field. This would enable us to compare the predictive value of the gene expression, mutational and cytogenetic compartments, and also help in the development of more precise risk stratification algorithms combining relevant information from the different biological layers.

In conclusion, we have validated the prognostic value of the Stellae-123 gene expression signature in adult and pediatric patients with AML. Our results indicate that this predictor is superior to the ELN-2017 risk stratification in adult patients, and that it also exhibits a good performance in pediatric patients. It becomes progressively evident that gene expression profiling and machine learning techniques can outperform conventional risk scores in the field of AML. The usefulness of Stellae-123 in order to inform about treatment strategies (e.g., alloHCT) and to test drug results in the context of clinical trials should be evaluated in the future.

## Data availability statement

Publicly available datasets were analyzed in this study. This data can be found here: Gene Expression Omnibus.

## Ethics statement

This study involving data from human participants was reviewed and approved by Comité de Ética Santiago/Lugo. The ethics committee waived the requirement of written informed consent for participation.

## Author contributions

AMO had the idea and performed the research. AMO, APR, and JDA analyzed the data and wrote the paper. BAR, MGP, MLR, CC, GM, AdlFB, PMF, MPE, analyzed the manuscript and made suggestions. All authors contributed to the article and approved the submitted version.

## Acknowledgments

We’d like to thank the Supercomputing Center of Galicia (CESGA) for their support. The content of this paper is part of the doctoral thesis of APR to obtain a PhD at the Department of Medicine, University of Santiago de Compostela.

## Conflict of interest

The authors declare that the research was conducted in the absence of any commercial or financial relationships that could be construed as a potential conflict of interest.

## Publisher’s note

All claims expressed in this article are solely those of the authors and do not necessarily represent those of their affiliated organizations, or those of the publisher, the editors and the reviewers. Any product that may be evaluated in this article, or claim that may be made by its manufacturer, is not guaranteed or endorsed by the publisher.

## Supplementary material

Supplementary Table 1 List of 69 genes included in Stellae-123.

Supplementary Table 2 Distribution of the different driver mutations in the BeatAML cohort according to the risk tertile assigned by Stellae-123.

Supplementary Table 3 Distribution of the different driver mutations in the AMLCG-8 cohort according to the risk tertile assigned by Stellae-123.

Supplementary Table 4 Distribution of the different driver mutations in the TARGET AML cohort according to the risk tertile assigned by Stellae-123.

Supplementary Figure 1 Outcomes of patients stratified according to the ELN-2017 criteria in the BeatAML **(A)** and AMCLG-2008 **(B)** cohorts, as we as those of pediatric patients in the TARGET AML cohort stratified according to the Clinical Risk Score **(C)**.

Supplementary Figure 2 Outcomes of pediatric patients (TARGET AML dataset) according to tertiles of expected survival predicted by Stellae-123 in the low-risk **(A)**, intermediate risk **(B)** and high risk **(C)** groups defined according to the Clinical Risk Score.
