# Corrigendum: Evaluation of the Stellae-123 prognostic gene expression signature in acute myeloid leukemia

**DOI:** 10.3389/fonc.2023.1302993

**Published:** 2023-10-04

**Authors:** Adrián Mosquera Orgueira, Andrés Peleteiro Raíndo, José Ángel Díaz Arias, Beatriz Antelo Rodríguez, Mónica López Riñón, Claudio Cerchione, Adolfo de la Fuente Burguera, Marta Sonia González Pérez, Giovanni Martinelli, Pau Montesinos Fernández, Manuel Mateo Pérez Encinas

**Affiliations:** ^1^Department of Hematology, University Hospital of Santiago de Compostela, Santiago de Compostela, Spain; ^2^Department of Hematology, Tomelloso Hospital, Ciudad Real, Spain; ^3^Unit of Hematology, IRCCS Istituto Romagnolo per lo Studio dei Tumori (IRST) “Dino Amadori”, Meldola, Italy; ^4^Department of Hematology, MD Anderson Cancer Center, Madrid, Spain; ^5^Department of Hematology, Hospital La Fe, Valencia, Spain

**Keywords:** leukemia, transcriptome, machine learning, survival, risk, prediction

In the published article, there was an error in affiliations 3 and 4. Instead of “^3^Department of Hematology, MD Anderson Cancer Center, Madrid, Spain, ^4^Unit of Hematology, IRCCS Istituto Scientifico Romagnolo per lo Studio e la Cura dei Tumori Istituto Tumori della Romagna IRST IRCCS, Meldola, Italy”, it should be “^3^Unit of Hematology, IRCCS Istituto Romagnolo per lo Studio dei Tumori (IRST) “Dino Amadori”, Meldola, Italy, ^4^Department of Hematology, MD Anderson Cancer Center, Madrid, Spain”.

The authors apologize for this error and state that this does not change the scientific conclusions of the article in any way. The original article has been updated.

