# Supplementary figures and images for: Evaluation of the Stellae-123 prognostic gene expression signature in acute myeloid leukemia
Source: Front Oncol. 2022 Aug 17;12:968340. doi: 10.3389/fonc.2022.968340 (PMC9428690; doi:10.3389/fonc.2022.968340)

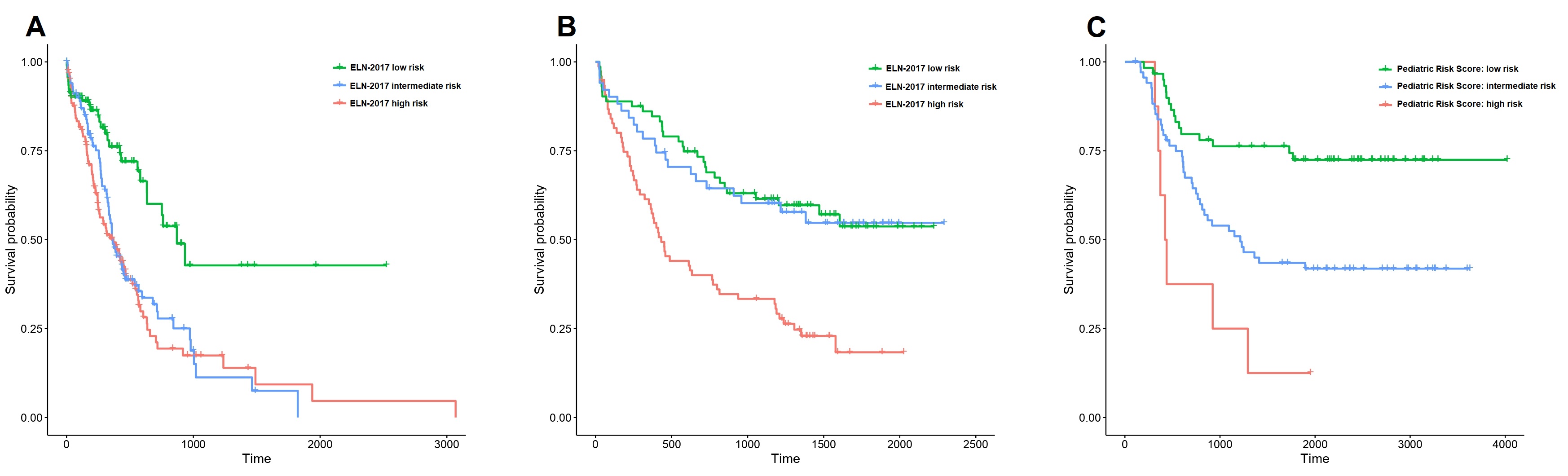

Supplement: Supplementary Figure 1 — Outcomes of patients stratified according to the ELN-2017 criteria in the BeatAML (A) and AMCLG-2008 (B) cohorts, as we as those of pediatric patients in the TARGET AML cohort stratified according to the Clinical Risk Score (C). [file Image_1.jpeg]

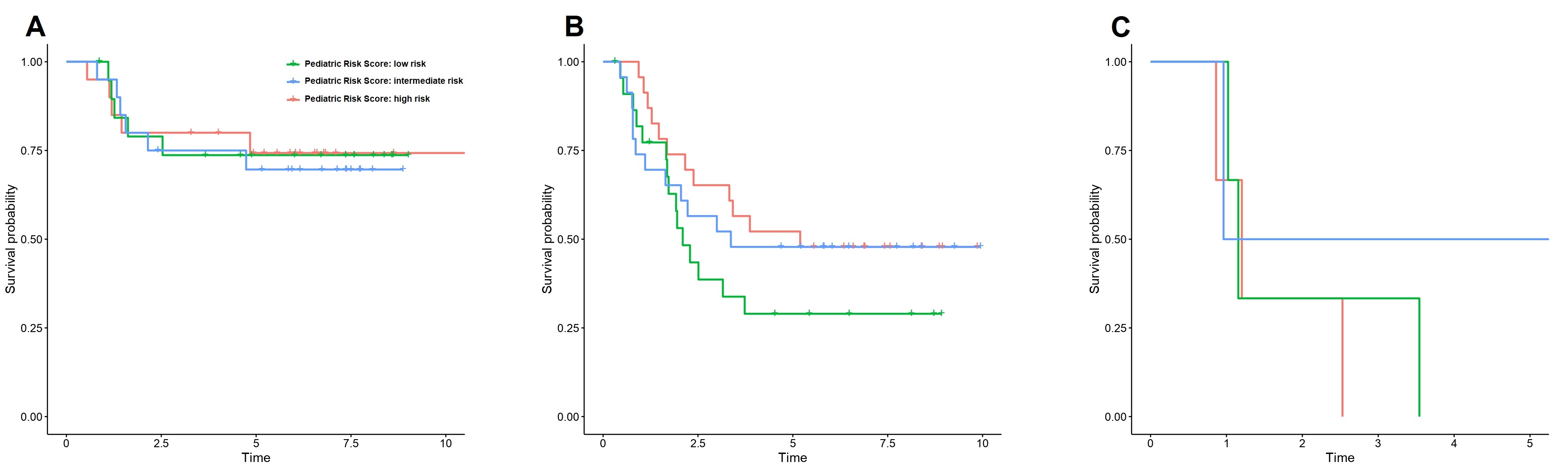

Supplement: Supplementary Figure 2 — Outcomes of pediatric patients (TARGET AML dataset) according to tertiles of expected survival predicted by Stellae-123 in the low-risk (A), intermediate risk (B) and high risk (C) groups defined according to the Clinical Risk Score. [file Image_2.jpeg]
